# Effect of Torso Kinematics on Gait Phase Estimation at Different Walking Speeds

**DOI:** 10.3389/fnbot.2022.807826

**Published:** 2022-03-30

**Authors:** Woolim Hong, Jinwon Lee, Pilwon Hur

**Affiliations:** ^1^J. Mike Walker '66 Department of Mechanical Engineering, Texas A&M University, College Station, TX, United States; ^2^School of Mechanical Engineering, Korea University, Seoul, South Korea; ^3^School of Mechanical Engineering, Gwangju Institute of Science and Technology, Gwangju, South Korea

**Keywords:** gait phase estimation, machine learning, torso variability, exoskeletons and prostheses, biomechanics

## Abstract

Human gait phase estimation has been studied in the field of robotics due to its importance for controlling wearable devices (e.g., prostheses or exoskeletons) in a synchronized manner with the user. As data-driven approaches have recently risen in the field, researchers have attempted to estimate the user gait phase using a learning-based method. Thigh and torso information have been widely utilized in estimating the human gait phase for wearable devices. Torso information, however, is known to have high variability, specifically in slow walking, and its effect on gait phase estimation has not been studied. In this study, we quantified torso variability and investigated how the torso information affects the gait phase estimation result at various walking speeds. We obtained three different trained models (i.e., general, slow, and normal-fast models) using long short-term memory (LSTM). These models were compared to identify the effect of torso information at different walking speeds. In addition, the ablation study was performed to identify the isolated effect of the torso on the gait phase estimation. As a result, when the torso segment's angular velocity was used with thigh information, the accuracy of gait phase estimation was increased, while the torso segment's angular position had no apparent effect on the accuracy. This study suggests that the torso segment's angular velocity enhances human gait phase estimation when used together with the thigh information despite its known variability.

## 1. Introduction

The gait cycle is a key concept in explaining human locomotion. The gait cycle commonly starts with heel-strike and ends with the next heel-strike of the ipsilateral leg (Alamdari and Krovi, 2017; Kawalec, 2017). A gait phase indicates the walking state (or progression) of the user within the gait cycle and estimating this user gait phase is crucial for controlling wearable assistive devices, such as powered prostheses (Gregg et al., 2014; Quintero et al., 2018; Hong et al., 2021; Lee et al., 2021) or exoskeletons (Kang et al., 2019; Seo et al., 2019; Sawicki et al., 2020). This is because wearable assistive devices should provide a synchronized motion with the user for stable walking, requiring an accurate user gait phase estimation (Gregg et al., 2014; Quintero et al., 2018; Kang et al., 2019; Seo et al., 2019; Sawicki et al., 2020; Hong et al., 2021; Lee et al., 2021). Conventionally, a discrete gait phase estimation (i.e., gait event detection) has been widely studied using different wearable sensor sets; several gait phase models have been proposed to separate the gait cycle into a different number of phases (Jasiewicz et al., 2006; Kotiadis et al., 2010; Abaid et al., 2013; Mannini et al., 2013; Allseits et al., 2017). Some researchers focused on heel-strike and toe-off detection with a rule-based algorithm using different sensor combinations (Jasiewicz et al., 2006; Allseits et al., 2017). Kotiadis et al. (2010) additionally detected the heel-off phase based on shank information. The hidden Markov model was also used to detect four different gait phases: heel-strike, flat-foot, heel-off, and toe-off (Abaid et al., 2013; Mannini et al., 2013). These discrete gait phase estimators could be used in the wearable device application to provide a synchronized motion control to the user.

Continuous gait phase estimation would be more effective in the seamless control of wearable devices since humans show continuously varying joint kinematics/kinetics trends (Rouse et al., 2014; Lee et al., 2016; Shorter and Rouse, 2018; Hong et al., 2019; Anil Kumar et al., 2020). Furthermore, for even more accurate gait phase estimation in a continuous manner, data-driven estimation techniques have recently evolved, utilizing diverse kinematics/kinetics information as an input dataset (Kang et al., 2019; Seo et al., 2019; Lee et al., 2021). Kang et al. (2019) achieved a neural network-based gait phase estimation relying on multiple sensors: encoders at the hip and IMUs at the thigh and torso. Seo et al. (2019) also implemented a recurrent neural network (RNN) model to estimate user gait phase using shank-mounted IMUs and additional foot pressure information for their model training. Lee et al. (2021) focused on angular positions and velocities of thigh and torso segments to estimate the user gait phase for their powered prosthesis application. As a result, they all achieved robust and accurate estimation in a continuous manner at different walking speeds (Kang et al., 2019; Seo et al., 2019; Lee et al., 2021). Even with those successful estimation results, the error rate varied according to walking speed. To be more specific, a larger deviation of error was found during the mid-stance phase in slow-walking (Lee et al., 2021). As suggested by Kang et al. (2019) and Lee et al. (2021), the torso movement exhibits a certain pattern during locomotion (Cappozzo, 1981; Thorstensson et al., 1984; Ceccato et al., 2009), thereby being used for estimating the gait phase. For instance, the torso maintains a particular forward inclination and oscillates around this position two times per gait cycle in the sagittal plane, and its rotation occurs one time per gait cycle in the horizontal plane (Ceccato et al., 2009). The torso information (e.g, segment's position and velocity), however, is also known to have high variability as per individual, and this variability becomes even higher in slow-walking (Thorstensson et al., 1984; Kerrigan et al., 2001; Dingwell and Marin, 2006; Asgari et al., 2015). We do not know yet whether this variability affects the estimation results at various walking speeds, especially at slow speeds.

Therefore, this article focuses on how torso information (i.e., segment's angular position and velocity) affects the accuracy of learning-based gait phase estimation at various walking speeds. We hypothesize that torso movement affects human gait phase estimation results at different walking speeds due to its known variability. To the authors' knowledge, the effect of the torso on the accuracy of gait phase estimation has not been spotlighted. In section 2, our gait phase estimation model is briefly explained. Also, the ablation study is described to identify the contribution of torso information to the estimation. In section 3, training results are presented and discussed based on the torso variability shown in the correlation matrix. To validate the proposed idea, prediction results are also shown in this section. We additionally present a heel-strike detection error for further evaluation. All the results are discussed and concluded in sections 4 and 5, respectively.

## 2. Methods

We previously proposed a speed-adaptive gait phase estimation model in Lee et al. (2021). Interestingly, it was found that gait phase estimation errors became larger during the mid-stance phase in slow-walking (Lee et al., 2021). In this study, we speculate on a possible remedy for this. The large estimation error may be because the torso deviates more while maintaining the balance in slow-walking (Dingwell and Marin, 2006; Asgari et al., 2015). This could be interpreted that torso kinematics may affect the estimation result. Therefore, we investigate the effect of torso kinematics on estimating the human gait phase by comparing the resulting estimations when torso information is included or excluded in model training.

### 2.1. Training Dataset

We utilized an open-source dataset, which can be found in Schreiber and Moissenet (2019), for our model training to guarantee a sufficient size of input data. This dataset included walkway walking data of 50 healthy subjects (26 male and 24 female) in five different speed conditions, such as *C*_1_: 0.0–0.4 m/s, *C*_2_: 0.4–0.8 m/s, *C*_3_: 0.8–1.2 m/s, *C*_4_: self-selected speeds (1.0–1.4 m/s), and *C*_5_: self-selected fast speeds (1.4–1.8 m/s). Fifty-two whole-body reflective markers were used to provide an individual's 3D motion information (Schreiber and Moissenet, 2019). We were able to generate the torso segment vector using the markers at the anterior-superior and posterior-superior iliac spine of both sides of the leg, and at the spinous process of the 10^*th*^ thoracic vertebrae. The thigh segment vector was generated using the markers at the greater trochanter and the lateral femoral epicondyle. We calculated the angular positions and velocities of the thigh and torso segments in the sagittal plane and utilized them for training our model. Furthermore, heel-strike and toe-off information could be estimated using ground reaction forces from two force plates. The data was sampled at 100 and 1.5 kHz for markers and force plates, respectively. Forty-two individuals' datasets were randomly selected for model training and validation, while the others were used for prediction.

### 2.2. Ground Truth Labeling

**Figure 1:**
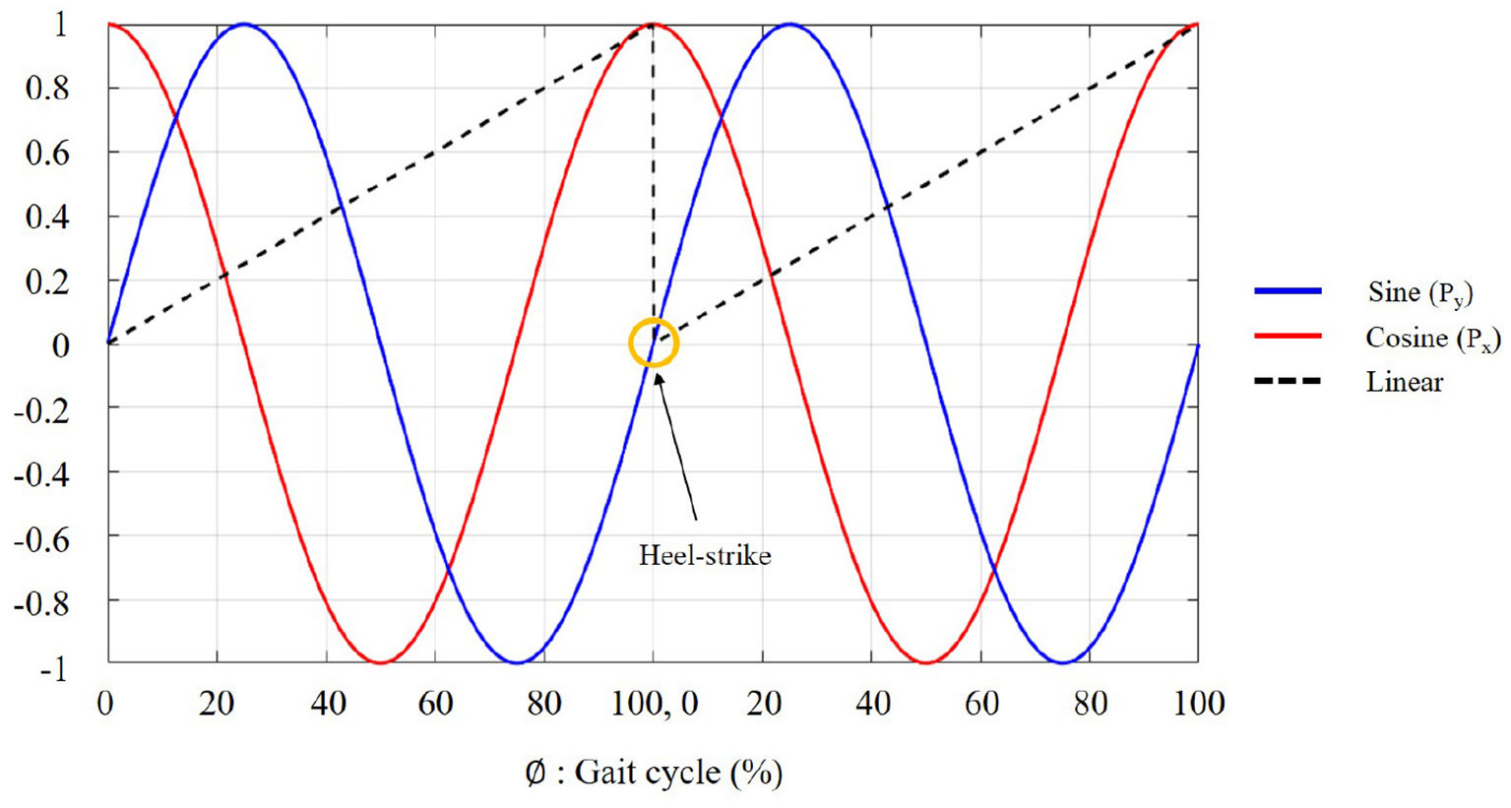
Ground truth labeling. Sine **(Blue)** and cosine **(Red)** functions are transformed into a bounded linear function **(Black)**. Gait initiation occurs at the heel-strike.

Heel-strike is conventionally used as a cue of gait initiation because the human gait cycle is usually defined from heel-strike to the next heel-strike on the same leg (Taborri et al., 2016; Vu et al., 2020). Based on the heel-strike, we labeled the data using a polar coordinate encoding method in the training session (Kang et al., 2019; Lee et al., 2021). This was because the nominal linear label is vulnerable to the discontinuity at heel-strike due to gait initiation (as shown in Figure 1), resulting in an undesired loss (i.e., mean-squared error) during model training. In Figure 1, ϕ refers to the percentage of the gait cycle, representing the user's walking progression between the heel-strikes, where ϕ∈[0, 100]. As shown in Equation (1), this walking progression (i.e., ϕ) can be mapped into θ during the entire gait cycle for the polar coordinate transformations, where θ∈[0, 2π]. By having two continuous sinusoidal functions as the ground truth (i.e., *P*_*x*_ and *P*_*y*_ in Equation (2), we could prevent the undesired error from the discontinuity at heel-strikes.


(1) $$\begin{array}{r} \begin{array}{c} \theta =\frac{2\pi }{100}\cdot \phi \end{array} \end{array}$$

(2) $$\begin{array}{r} \left(P_{x},P_{y}\right)=\begin{array}{c} \left(\cos\theta ,\sin\theta \right) \end{array} \end{array}$$

Following Equations (3) and (4), those sine and cosine functions can be transformed into a linear function $\hat{\tau }$, which is bounded in [0,1], representing the continuous gait phase. This linear gait phase estimation is usually utilized for controlling wearable devices (Kang et al., 2019; Hong et al., 2021).


(3) $$\begin{array}{r} \tau =\frac{1}{2\pi }atan2\left(Py,Px\right) \end{array}$$

(4) $$\hat{\tau }=\{\begin{array}{cc} \tau & P_{y}\geq 0\\ \tau +1 & P_{y}<0 \end{array}$$

### 2.3. Neural Network

**Figure 2:**
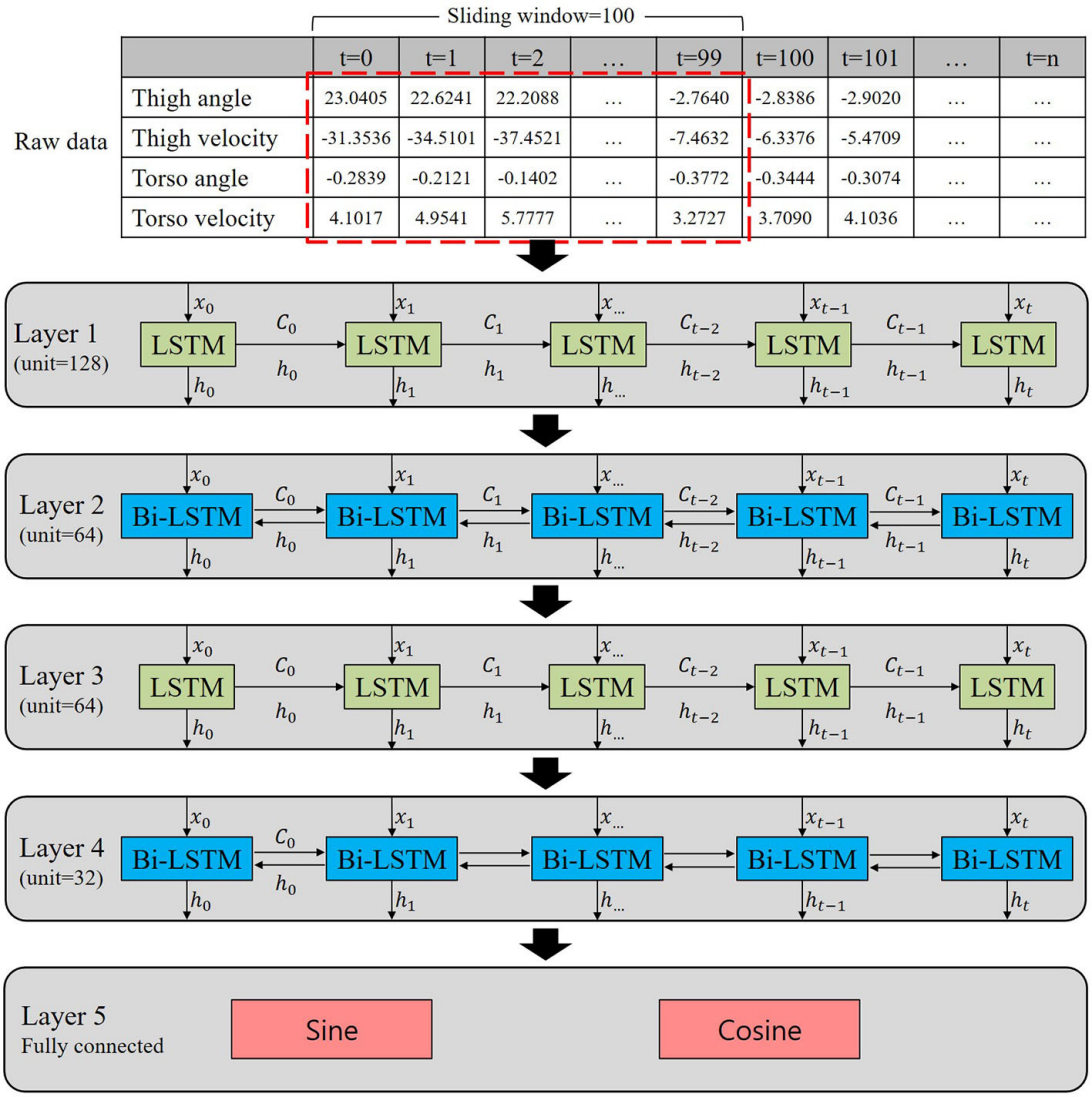
Proposed neural network architecture for the gait phase estimation. The network consists of five layers with long short-term memory (LSTM) and bidirectional LSTM (Bi-LSTM). Layer 1–4 has 128, 64, 64, and 32 units, respectively. Layer 5 is fully connected and results in the output as sine and cosine functions.

Torso movement is known to have higher variability compared to thigh movement during walking. Even though highly variable signals may have the potential for more information, we still do not know whether the torso information enhances the gait phase estimation accuracy. In order to investigate the contribution of torso information to gait phase estimation, an ablation study was performed in this study using torso angular position and velocity. We prepared four input datasets for model training: (Set 1) angular positions and velocities of thigh and torso segments; (Set 2) angular position and velocity of the thigh segment and angular velocity of the torso segment; (Set 3) angular position and velocity of the thigh segment and angular position of the torso segment; and (Set 4) angular position and velocity of the thigh segment. As shown above, both the thigh segment's angle and velocity were always included in the four datasets, while the torso segment's angular position and velocity conditions were changed. Also, three different speed conditions (e.g., *C*_2_, *C*_2_−*C*_5_, and *C*_3_−*C*_5_) were given for the model training to be generalized to diverse walking speeds. The trained model only utilizing *C*_2_ was named the *slow model*, while the models using *C*_2_−*C*_5_ and *C*_3_−*C*_5_ were called the *general model* and *normal-fast model*, respectively. *C*_1_ was excluded because it referred to extremely slow speeds. A long short-term memory (LSTM) was utilized in this study due to its powerful performance with chronological data, such as time series prediction (Hochreiter, 1997; Kang et al., 2019; Lee et al., 2021). Further, bidirectional LSTM (Bi-LSTM) was implemented to achieve both forward and backward learning during the training process (Graves, 2005). This allowed the given model to learn from past and future information. The size of the sliding windows for the model was chosen to be 100, which was deemed to be appropriate for the collected data with relatively short lengths. Figure 2 shows the proposed network architecture. Our network consists of five layers with LSTM and Bi-LSTM. Layer 1–4 has 128, 64, 64, and 32 units, respectively. As depicted in Figure 2, the current input (*x*_0_) updates the cell state (*C*_0_) and the output (*h*_0_). The cell state updates the information from input data and transfers the previously learned information to the next block. Layer 5 results in the output as the sine and cosine functions, as explained in section 2.2. We selected the last value in the sequence to get the gait phase at time *t*. The network model was trained with the Adam optimizer and mean-squared error (MSE) was used as a loss function with a batch size of 64. To prevent the over-fitting, the model was trained for a maximum of 100 epochs, stopping early if the validation loss did not continue to decrease in 10 epochs.

### 2.4. Statistical Analysis

Statistical analysis was performed to determine the significance of the torso information in the gait phase estimation model using RStudio statistical software (RStudio ver. 1.3.1093). For model training, we used three models (i.e., general, slow, and normal-fast models) with four input datasets (i.e., Sets 1–4). For the training results, we performed a two-way ANOVA to identify the effects of the training dataset (i.e., Sets 1–4) and three different models (i.e., general, slow, and normal-fast). For the prediction results, three two-way ANOVAs (each for a speed-dependent model) were performed to examine the effect of the trained dataset (i.e., Sets 1–4) and the speed condition (*C*_2_−*C*_5_). We performed another two-way ANOVA for the heel-strike detection error to identify the effect of the dataset and the speed condition. In a multiple comparison, Bonferroni correction was used as a *post-hoc* test. A significance level of 0.05 was used in all analyses. Throughout this article, the statistical significance was symbolized as follow: * = *p* ≤ 0.05, ** = *p* ≤ 0.01, and *** = *p* ≤ 0.001.

## 3. Results

Throughout this section, a total of 12 conditions are given based on four different training sets (i.e., Sets 1–4) and three different speed-dependent models: general, slow, and normal-fast. We performed a training process for each condition and collected the final loss-value (i.e., MSE) for each independent model.

### 3.1. Training Results

**Figure 3:**
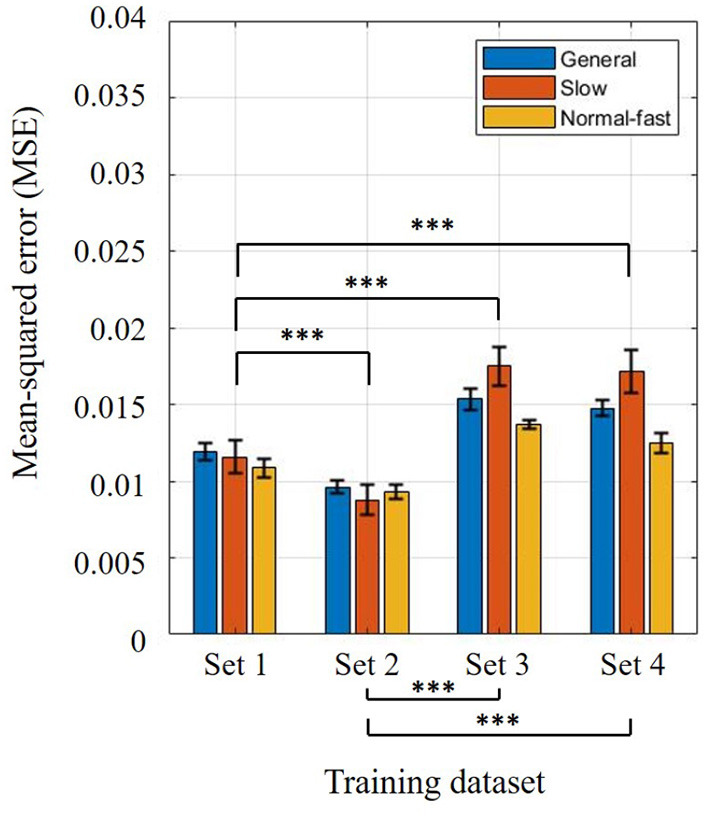
Training results using four different training sets: **(Set 1)** angular positions and velocities of thigh and torso segments, **(Set 2)** angular position and velocity of thigh segment, and torso segment angular velocity, **(Set 3)** angular position and velocity of thigh segment, and torso segment angular position, **(Set 4)** position and velocity of thigh segment. Bar colors correspond to three different trained models: general, slow, and normal-fast walking. Bar graphs and error bars correspond to the mean and ± 1SD.

The two-way ANOVA for the training results found that both training sets (*p* <0.001) and three different models (*p* <0.001) were significant. Training results using four different training sets (Sets 1–4) were compared to investigate the torso information effect on the estimation (see Figure 3). Note that Set 4 was considered as the baseline because it only contained thigh information. As shown in Figure 3, there was no significant difference when the torso angle was included compared to Set 4 (Set 3 vs. Set 4, *p* = 0.052). The highest accuracy was found when position and velocity of the thigh segment and torso segment velocity were utilized for model training (Set 2, *p* <0.001), while the second-highest accuracy was achieved with both angular positions and velocities of the thigh and torso segments (Set 1, *p* <0.001). Between Sets 1 and 2, the error increased when the torso angle was included (*p* <0.001). On the other hand, the estimation errors were reduced when the torso angular velocity was included in the training set (Sets 1 and 2 vs. Sets 3 and 4, *p* <0.001). This implies that the contribution of torso angular position and velocity may differ. Considering the speed-dependent model conditions, it was obvious that the slow model shows the highest error in Sets 3 and 4 (*p* <0.001). To identify a link between torso variability and gait phase estimation results, we additionally checked how much deviations the thigh and torso have per individual at different walking speeds.

**Figure 4:**
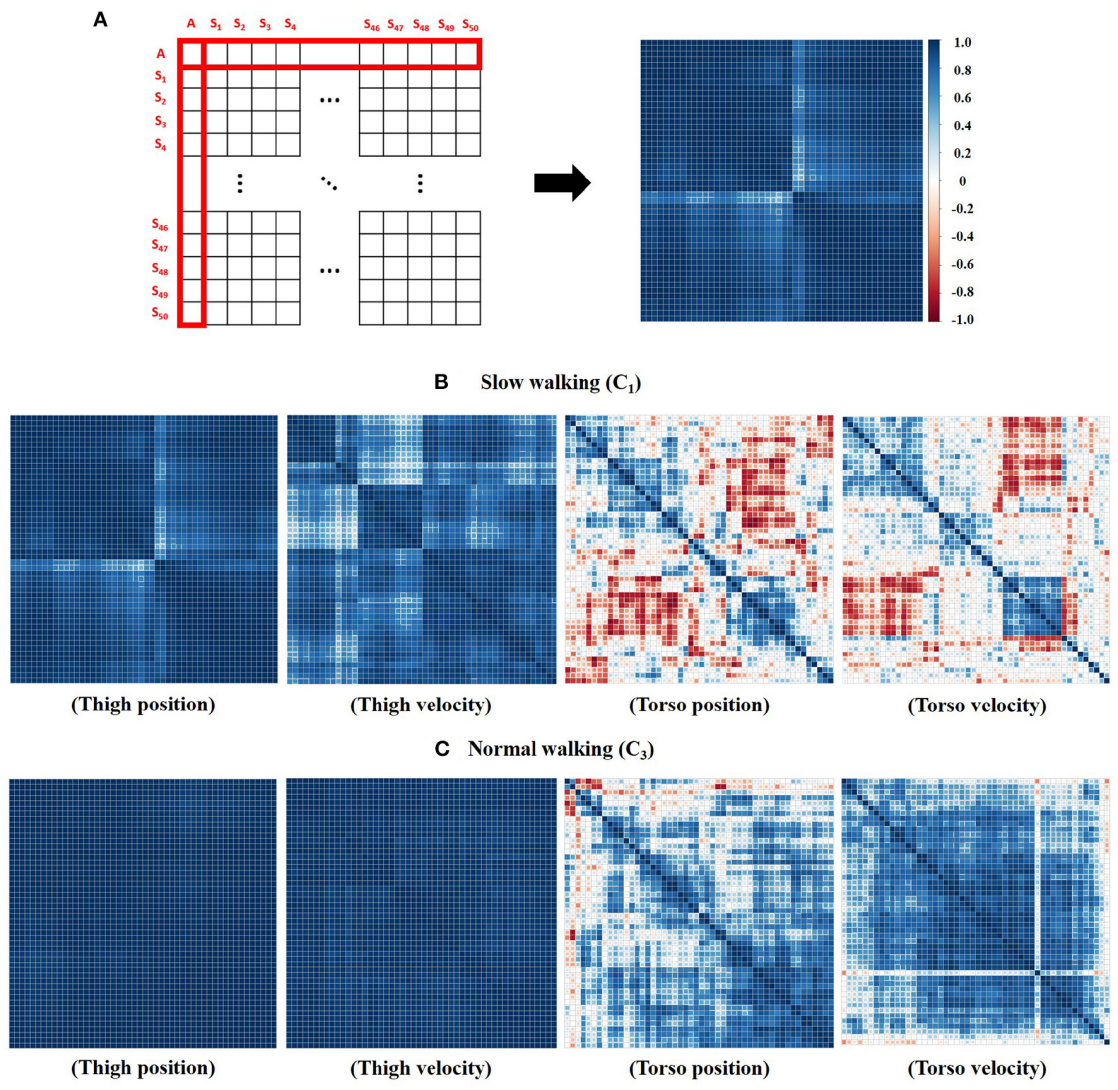
Correlation matrix. **(A)** 51 ×51 dimension of the correlation matrix. The color map indicates the correlation coefficient value: blue (positive), red (negative), and white (≃ 0). **(B)** The correlation results of slow-walking data (*C*_1_). **(C)** The correlation results of normal-walking data (*C*_3_).

As depicted in Figure 4A, the correlation matrix was generated using 51 variables, having 51 ×51 dimensions. The given variable set consists of a single mean trajectory of all subjects (*A*) and each subject's mean trajectory (*S*_*i*_, where *i* refers to subject id). Each cell in the matrix shows the correlation between two variables. Our matrix starts with the mean trajectory of all subjects (*A*) and ends with the 50th subject's mean trajectory (*S*_50_). So, we could only focus on the first row or column (red box in Figure 4A) to see the correlation between each individual's trajectory (*S*_1, 2, ..., 49, 50_) and the mean trajectory of all subjects (*A*). The mean and SD of those correlation coefficients are presented in Table 1. According to Table 1, both the thigh and the torso show the highest variability in slow-walking (*C*_1_), which is consistent with other studies (Dingwell and Marin, 2006; Asgari et al., 2015). The torso correlations are specifically smaller than those of the thigh. Compared to the normal and fast speed conditions, the slow walking data (i.e., C1 and C2) showed significantly higher variability for both thigh and torso. The torso data was specifically more sensitive to the walking speed according to Table 1. Even at the normal walking (i.e., the highest correlation result), the torso shows less correlation (e.g., position: 0.7032 ± 0.2546, velocity: 0.7996 ± 0.2011 in *C*_3_) when the thigh correlation is close to 1 (e.g., position: 0.9925 ± 0.0057, velocity: 0.9835 ± 0.0085 in *C*_3_). Between torso angular position and velocity, the torso velocity shows a higher correlation per individual than the torso position throughout all speed conditions. In Figure 4, the correlation matrix is also illustrated using a colormap. Figures 4B,C depict the correlation matrix of thigh and torso information at two different walking speeds: *C*_1_ and *C*_3_. It is obvious that thigh information shows a much higher correlation with each other than torso information in both slow and fast walking (as shown in Figures 4B,C). It is also apparent that faster walking speed (Figure 4C) shows a higher correlation than slower walking speed (Figure 4B) for all information.

**Table 1:** Mean and SD of correlation coefficients for each dataset in five different speed conditions.

**Walking**	**Thigh position**	**Thigh velocity**	**Torso position**	**Torso velocity**
**speed**	**(mean ± 1SD)**	**(mean ± 1SD)**	**(mean ± 1SD)**	**(mean ± 1SD)**
C_1_	0.9663 ± 0.0382	0.9059 ± 0.0656	0.1259 ± 0.3269	0.2222 ± 0.2817
C_2_	0.9892 ± 0.0074	0.9726 ± 0.0169	0.4092 ± 0.5476	0.4936 ± 0.5278
C_3_	0.9925 ± 0.0057	0.9835 ± 0.0085	0.7032 ± 0.2546	0.7996 ± 0.2011
C_4_	0.9917 ± 0.0096	0.9824 ± 0.0063	0.6638 ± 0.3098	0.7630 ± 0.2857
C_5_	0.9941 ± 0.0047	0.9876 ± 0.0063	0.5643 ± 0.3098	0.6999 ± 0.2857

*The correlation results were calculated between each subject's trajectory (S_i_) and the mean trajectory of all subjects (A)*.

### 3.2. Prediction Results

**Figure 5:**
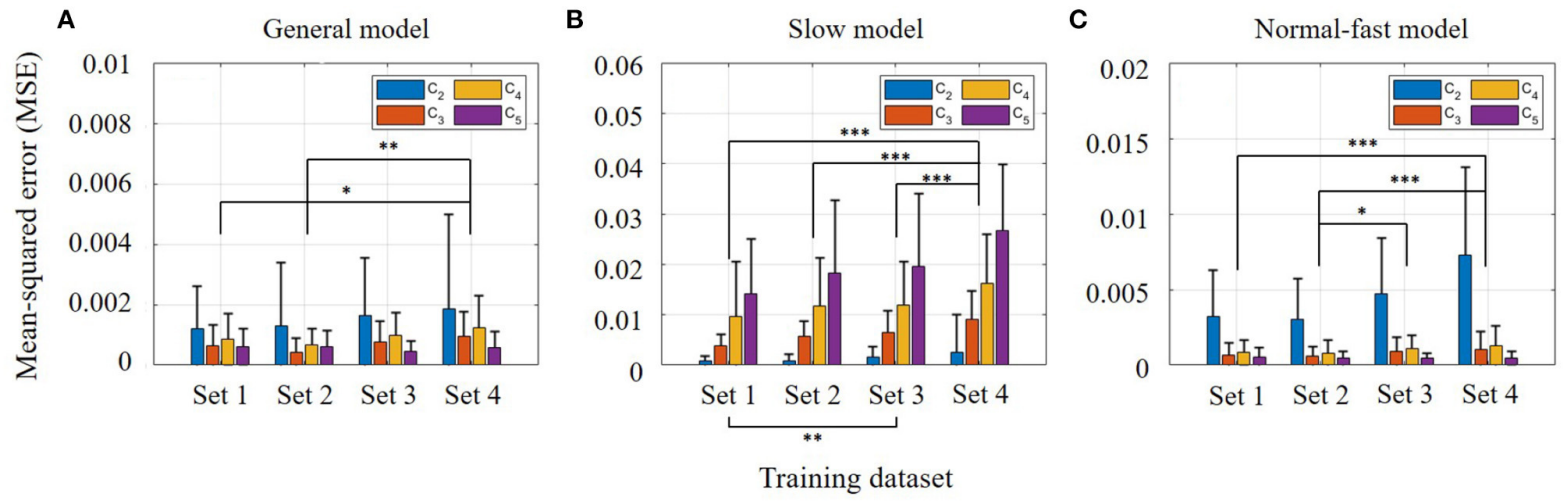
Prediction results from three trained models: **(A)** General model using *C*_2_−*C*_5_. **(B)** Slow model using only *C*_2_ **(C)** Normal-fast model using *C*_3_−*C*_5_. Bar graphs and error bars correspond to the mean and + 1SD. Bar colors correspond to walking speeds: *C*_2_−*C*_5_.

A two-way ANOVA was performed for each speed-dependent model (i.e., general, slow, and normal-fast models) to identify the significance of the trained dataset (i.e., Sets 1–4) and the speed conditions (i.e., *C*_2_−*C*_5_). The prediction process was performed using Sets 1–4. Data from eight subjects were randomly selected to be used for the prediction. Also, individuals' walking data at four different speeds (i.e., *C*_2_−*C*_5_) were used for evaluating the prediction results. The prediction errors are described in Figure 5 to identify the torso kinematics effect on the gait phase estimation. Figure 5A shows the prediction result of the general model. In this model, both training sets (*p* = 0.002) and speed conditions (*p* <0.001) were significant. Figures 5B,C show the results of the slow model and the normal-fast model, respectively. In the cases of the slow model and the normal-fast model, both models also showed the significant effects of the training sets (*p* <0.001) and the speed conditions (*p* <0.001) according to each two-way ANOVA. The estimation error specifically increased when the walking speed became faster in the slow model (*p* <0.001). On the other hand, in the normal-fast model, the highest error was observed at slow walking speed (*p* <0.001). In general, Figure 5A shows the best estimation result while covering all different speed conditions (i.e., *C*_2_−*C*_5_). The relatively high errors were still shown at slow speeds due to the high variability of the dataset in slow-walking. This could be further explained by comparing the results of each dataset. In Figure 5A, there was no significant interaction effect between the prediction dataset and walking speed. Among the given datasets, Set 4 showed the highest error in the prediction compared to Sets 1 (*p* = 0.0438) and 2 (*p* = 0.0042). Set 3 had no significant difference from Set 4. According to the *post-hoc* test based on speed conditions, all of them showed a significant difference from each other, except *C*_3_ and *C*_5_.

**Figure 6:**
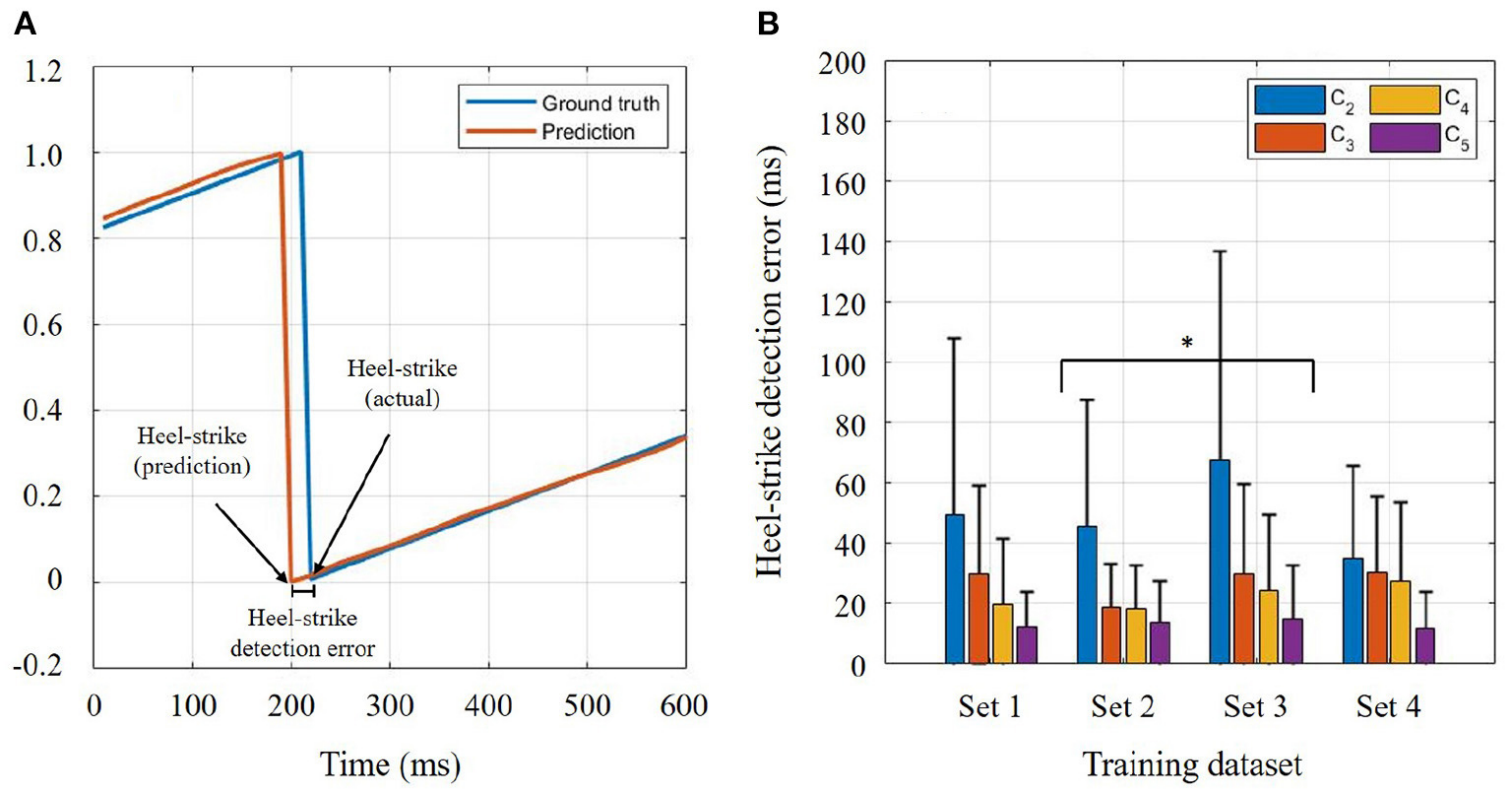
Heel-strike detection error. **(A)** Temporal difference between actual heel-strike and predicted heel-strike. **(B)** Heel-strike detection error in four different speed conditions. The general model was chosen for the comparison. Bar graphs and error bars correspond to the mean and + 1SD. Bar colors correspond to walking speeds: *C*_2_, *C*_3_, *C*_4_, and *C*_5_.

Another two-way ANOVA was performed to identify the effects of training sets and walking speeds on the heel-strike detection errors in the general model. This error refers to the temporal difference between actual heel-strike and predicted heel-strike (as shown in Figure 6A). The two-way ANOVA for heel-strike prediction found that both training sets (*p* = 0.046) and speed conditions (*p* <0.001) were significant. According to the *post-hoc* comparison, only Sets 2 and 3 were significantly different (*p* = 0.044) in the training set condition. In Figure 6B, *C*_2_ showed the highest error in the heel-strike detection (*p* <0.001) compared to the other speed conditions. At the fast speed, the detection error was significantly reduced compared to *C*_3_ (*p* = 0.002), but it was not significant compared to *C*_4_ (*p* = 0.076). There was no significant difference between *C*_3_ and *C*_4_.

## 4. Discussion

It is no doubt that thigh information is a key factor for human gait phase estimation (Quintero et al., 2018; Kang et al., 2019; Seo et al., 2019; Hong et al., 2021; Lee et al., 2021). Across all walking speeds, its robustness can also be shown in Table 1. On the other hand, it is obvious that the torso information has higher variability compared to the thigh information during walking. Owing to the fact that the torso segment's angular position showed even higher variability than the torso segment's angular velocity, we could assume that torso position information may hinder a successful estimation of the user's gait phase. Assuming Set 4 as a baseline, Sets 3 and 4 comparison tells the isolated effect of torso segment's angular position on gait phase estimation. To be more specific, when the torso position information was solely utilized with the thigh information, no apparent effect was found in the prediction result according to Figure 5. Likewise, the effect of the torso segment's angular velocity can be identified by the comparison between Sets 2 and 4. Unlike the torso position information, significant error reductions were found in predicting the user's gait phase when torso velocity information was included in the training set. This implies that the torso segment's angular velocity is beneficial to gait phase estimation despite its relatively high variability compared to thigh information (as shown in Table 1). However, the heel-strike detection error result showed a different trend in the slow walking speed condition. Compared to Set 4, the heel-strike detection error became greater at slow speed (*C*_2_) when any torso information was contained. This may imply that heel-strike detection is more sensitive to torso variability in slow-walking. Higher torso variability at slow speed (Dingwell and Marin, 2006) may hinder the accurate detection of the heel-strike. For other speeds, torso velocity information also showed a beneficial effect on gait phase estimation.

As we mentioned in section 2.3, we did not have much choice in the size of the sliding window for our model training. Since, the chosen dataset was collected on a walkway, it contained relatively short time-series data (compared to treadmill walking), including only a single gait cycle at most. We considered an alternative dataset, but the selected dataset contained an abundant number of subjects, which guaranteed to show individuals' variability. The chosen window size may affect the estimation accuracy, but we obtained sufficiently high accuracy in our estimation. To be fair with validating this claim, we implemented the same window size (i.e., 100) as our previous model (Lee et al., 2021) and compared its training results (i.e., MSE) to this study. As a result, there was no significant difference between them (Lee et al., 2021; 1.10E-02 vs. this study: 1.19E-02), thereby alleviating the concern about the window size. Furthermore, compared to Lee et al. (2021), we improved the estimation accuracy during the mid-stance phase at slow walking speed (i.e., *C*_2_). In this work, we computed the mean-squared error during 30–50% of the gait cycle and compared it to the result of Lee et al. (2021). The prior model, Lee et al. (2021), yielded 9.88E-04 ± 8.47E-04, while the proposed model yielded 5.34E-04 ± 7.11E-04 in this study.

In future work, the authors plan to develop a user-adaptive gait phase estimator for enhancing an individual's gait trait adaptability. This is important for providing user-specific control of wearable devices based on user-specific gait estimation. This is because all individuals have their own gait traits, considering these traits is expected to give a better estimation of the individual. Also, we plan to implement a convolutional neural network (CNN) with LSTM to obtain faster estimation. The proposed method will be implemented to control a custom-built powered prosthesis. The authors have controlled the powered prosthesis using a phase variable, deriving from the user's thigh motion (Hong et al., 2021). Unlike the phase variable, a learning-based gait phase estimation utilized a plentiful dataset, so we could expect improved robustness, leading to more stable control of the prosthesis.

## 5. Conclusion

Torso information has been used for estimating the human gait phase, but its effect on the gait phase estimation has not been studied so far. We investigated the torso segment information effect by comparing the estimation results using four different datasets (i.e., Sets 1–4). As a result, the torso segment's angular velocity supported an accurate gait phase estimation for all walking speeds despite its relatively high variability compared to thigh information. On the other hand, the torso segment's angular position had no significant effect on the accurate estimation. As walking speed became slower, the torso variability increased, and lower accuracy was obtained. This study, therefore, showed the torso segment's angular velocity is more beneficial than the torso segment's angular position for gait phase estimation.

## Data Availability Statement

The datasets and the main code utilized for this study can be found in the GitHub repository for future development: https://github.com/ulim88/Frontiers-TorsoEffect.

## Author Contributions

WH is the primary contributor for the proposed concept, data analysis, and methodology. JL contributed for data analysis and methodology. PH served as the principal investigator. All authors contributed to writing and reviewing this article.

## Conflict of Interest

The authors declare that the research was conducted in the absence of any commercial or financial relationships that could be construed as a potential conflict of interest.

## Publisher's Note

All claims expressed in this article are solely those of the authors and do not necessarily represent those of their affiliated organizations, or those of the publisher, the editors and the reviewers. Any product that may be evaluated in this article, or claim that may be made by its manufacturer, is not guaranteed or endorsed by the publisher.
